# Novel genetic matching methods for handling population stratification in genome-wide association studies

**DOI:** 10.1186/s12859-015-0521-4

**Published:** 2015-03-14

**Authors:** André Lacour, Vitalia Schüller, Dmitriy Drichel, Christine Herold, Frank Jessen, Markus Leber, Wolfgang Maier, Markus M Noethen, Alfredo Ramirez, Tatsiana Vaitsiakhovich, Tim Becker

**Affiliations:** 1 German Center for Neurodegenerative Diseases (DZNE), Sigmund-Freud-Str. 25, Bonn, 53127 Germany; 2 Institut für Medizinische Biometrie, Informatik und Epidemiologie, Universität Bonn, Sigmund-Freud-Str. 25, Bonn, 53127 Germany; 3 Abteilung für Psychiatrie und Psychotherapie, Universitätsklinikum Bonn, Sigmund-Freud-Str. 25, Bonn, 53127 Germany; 4 Institut für Humangenetik and Life & Brain Center, Universität Bonn, Sigmund-Freud-Str. 25, Bonn, 53127 Germany

**Keywords:** Genome-wide association studies, population stratification, genetic matching, structured association

## Abstract

**Background:**

A usually confronted problem in association studies is the occurrence of population stratification. In this work, we propose a novel framework to consider population matchings in the contexts of genome-wide and sequencing association studies. We employ pairwise and groupwise optimal case-control matchings and present an agglomerative hierarchical clustering, both based on a genetic similarity score matrix. In order to ensure that the resulting matches obtained from the matching algorithm capture correctly the population structure, we propose and discuss two stratum validation methods. We also invent a decisive extension to the Cochran-Armitage Trend test to explicitly take into account the particular population structure.

**Results:**

We assess our framework by simulations of genotype data under the null hypothesis, to affirm that it correctly controls for the type-1 error rate. By a power study we evaluate that structured association testing using our framework displays reasonable power. We compare our result with those obtained from a logistic regression model with principal component covariates. Using the principal components approaches we also find a possible false-positive association to Alzheimer’s disease, which is neither supported by our new methods, nor by the results of a most recent large meta analysis or by a mixed model approach.

**Conclusions:**

Matching methods provide an alternative handling of confounding due to population stratification for statistical tests for which covariates are hard to model. As a benchmark, we show that our matching framework performs equally well to state of the art models on common variants.

**Electronic supplementary material:**

The online version of this article (doi:10.1186/s12859-015-0521-4) contains supplementary material, which is available to authorized users.

## Background

Genome-wide association studies (GWAS), see e.g. [1,2] and references therein, have been proven to be useful to detect genetic risk variants that are involved in the etiology of complex diseases. Nonetheless, common single nucleotide polymorphisms (SNPs) do not account for the total inherited risk of complex diseases. One cause might be attributed to uncommon or rare variants, which are progressively covered by larger DNA micro-arrays and Next Generation Sequencing. A large sample size is required in order to obtain sufficient statistical information to detect possible effects. It has been recommended by the authors of [3,4] to extent GWAS and sequencing studies to admixed ancestral populations. The hope is to narrow down linkage disequilibrium (LD) blocks by probing additional genetic variation. For the combined analysis of rare variants and admixed populations it becomes more complicated to correct for confounding effects: on the one hand, individuals showing the same rare variants may originate from distinct ancestral populations and therefore differ in effect sizes and prevalences. On the other hand, individuals sharing ancestry may have non-trivial local differences due to drift, selection or partial admixture. The task is to implement a chromosomal region-specific matching [3,5] in order to overcome the problem of complex confounding effects rising from population structure when considering rare and common variants.

*Population stratification*, the characteristic of a population sample to enable the occurrence of spurious statistical findings, rises as a consequence of diverging allele frequencies in combination with varying phenotype prevalences. Note that population structure, that is the mere existence of diverging allele frequencies caused by population subgroups [6,7], is insufficient to create confounding by itself [8]. The main attribute of population stratification is the statistically significant but spurious association [9], that results in an inflated type-1 error rate (false-positives). The reason is, that due to confounding the distribution function for the test statistic is biased in a virtually unpredictable way. Using the known unbiased distribution function regardless will consequently provide misguided P-values. It is worth noticing that this may simultaneously cause inflation of the type-2 error rate (power-loss) or leads to biased estimates of effect sizes. To account for population stratification, a lot of effort has been put into the development of approaches during the last two decades:

*Genomic control* (GC) [10,11] has been widely accepted as a method which provides a measure for the extend of genomic inflation. A genome-wide inflation factor is calculated and used to rescale the test statistics for residual bias. This is done under the assumption that virtually every SNP is in null-association with the trait and the more fragile assumption that the degree of inflation is constant across the genome. It should be stressed that under polygenic inheritance the genomic inflation strongly depends [12] on properties like heritability, number of causal variants, LD distribution and sample proportions. In [13,14] it has been shown that GC hardly provides a sufficient tool to correct for population structure, but is useful to correct for residual inflation.

The *structured association* [15] approach relies on assigning sample individuals to population clusters. Testing for associations is then performed relying on cluster information [16,17]. Usually clustering algorithms incorporate assumptions on the underlying populations, i.e. they are model-dependent [16,18], or they are based on the results of principal component approaches [19].

The basic idea of the *principal components* (PC) [20,21] approach is to reduce the number of dimensions with a minimal loss of information. It is based on decomposing a matrix, whose entries quantify genetic properties of the individuals to eigenvectors and eigenvalues. Leading components are then used as regression covariates in the association model or for randomization tests [22]. PCs are widely accepted and employed, but may also have drawbacks: the decomposition is sensitive to outlying individuals [23,24] and genome-wide data are required. The power of regression models may suffer from a large number of parameters. Additionally, important information about population substructure may be hidden in sub-leading components. Prominent PC approaches are the *principal components analysis* (PCA) basing on eigen-decomposition [25] or singular value decomposition [26] of the genotype matrix. Another is *multidimensional scaling* (MDS) [27], where the identity-by-state (IBS) matrix is linearly projected down on those axis where the data have the largest extend.

There are also genetic optimal matchings using the results of the PCA approaches [28] or based on genetic similarity scores [29] or stratification scores [30].

Most recently *linear mixed models* (LMM) [31,32] has made its impact in statistical genetics. These models comprise a fixed effect and a random effect term, where polygenic effects and effects due to sample structure are modeled into the random part. LMM have proven to be quite successful in handling population structure in aggregation with family structure and cryptic relatedness [33-35]. It has also been advised, to include population structure in terms of PCA as a fixed effect [36].

In this work we provide an optimal population matching based on a genetic similarity score. We use the pairwise IBS matrix as genetic similarity score, which can optionally be calculated genome-wide or locally. We develop a set of structuring strategies based on the assignment algorithm in terms of matched case-control pairs and matched groups with at least one case and one control. We also review on the Hungarian Clustering Algorithm [37] that is employed in order to obtain a population clustering without model assumptions. These matching strategies are supplemented by validation methods which serve for quality controlling the found case-control matches. Our work is related to that of [29], but instead of using a matching algorithm that resides on several degrees of freedom, we apply our validation methods on the results and eventually re-run the bipartite matching algorithm on invalid matches. We also develop a particularization of the Cochran-Armitage trend (CAT) test [38,39] to the group structures and will compare both modified and unmodified tests with a principal components approach.

## Methods

### Genetic similarity score

We introduce the genetic similarity score matrix *s*. Its components *s*_*ij*_ measure the genetic relatedness between two individuals *i* and *j*. In this work we choose the averaged IBS value for the genetic similarity score, which is given by the portion of alleles shared by state in both individuals:

(1) $$ s_{ij} = 1 - \frac{1}{2N} \sum_{k=1}^{N} |g_{ik} - g_{jk}| ~,  $$

where *N* is the number of loci successfully genotyped in both individuals and *g*_*ik*_ is the nominal genotype {0,1,2} (number of expressed minor alleles) for individual *i* at locus *k*. It is also possible to weight certain loci, for instance based on Hardy-Weinberg equilibrium or minor allele frequency [29]. The determination of the genetic similarity score matrix is computationally expensive. In order to expedite the calculation we store genotypes in a binary encoding, see Additional file 1: Appendix A, and employ modern versions of the Hamming weight method [40] to count coinciding alleles. In our implementation the calculation of the genetic similarity score using the binary encoding is roughly 15 times faster compared to a byte-wise storage with byte-wise arithmetics.

### Structuring strategies

The basic idea is to divide the study sample into genetically similar case-control pairs in an overall optimal way. The measure for this similarity is given by the genetic similarity score matrix *s* of eq. (1). Finding pairs from two distinct sets (here cases & controls), whose elements of both sets are connected by weights, in an optimal way is well-known as the *weighted assignment problem* [41]. The Kuhn-Munkres *“Hungarian” Method* [42], which is the graph theoretical solution of a ’maximum weighted bipartite matching’, solves this problem in polynomial time [43]. Algorithms available today are of complexity $\mathcal {O}(n^{3})$ [44,45]. For a detailed modern illustration of the method see [46]. In the following we introduce a set of structuring strategies that are based on this bipartite matching.

#### Case-control pairwise matching

The Hungarian Method requires balanced and complete bipartite graphs. Therefore, in case of different cardinalities of the sets of controls *O* and cases *A* we extend the smaller set to have max(|*O*|,|*A*|) cardinality. We add $\left ||O|-|A|\right |$ additional elements (“*sinks*”) to the smaller set. Then we consider the balanced bipartite graph *G*(*V*,*E*,*w*) with *vertices* *V*=*O*∪*A*, *O*∩*A*=*∅*, *edges* *E*⊆*O*×*A* and *weights* $w:E\rightarrow \mathbb {R}$. The weights are given by the genetic similarity score of eq. (1), *w*(*i*,*j*)=*s*_*ij*_. For the edges which are incident upon sinks the weights are set to zero. The Hungarian Method returns a matching *M*⊂*E* with max(|*O*|,|*A*|) matched pairs. From that we remove matches comprising sinks. Thus, the individuals that are matched to the sinks are also removed, ending up with min(|*O*|,|*A*|) cases and controls, respectively. By doing this, we reduce the sample size to an equal number of cases and controls. Subsequently, we perform a stratum validation as described in section ‘Stratum validation strategies’.

#### Case-control groupwise matching

In order not to reduce asymmetric samples (|*O*|≠|*A*|) too extensively, and therefore lose power, one can refit unmatched individuals into the sample. To achieve that, we perform an initial matching as described in section ‘Case-control pairwise matching’. Afterwards we repeat the matching process between matched cases with unmatched controls (those that were removed) and vice versa. The newly matched individuals are added to the case-control pairs of the initial matching. Thus, instead of receiving min(|*O*|,|*A*|) case-control pairs, we end up with min(|*O*|,|*A*|) small groups of at least one case and one control. The process of re-matching unmatched individuals to matched individuals is repeated until either every individual is matched or until no individual is successfully been re-matched during a single run of the re-matching process. The latter may happen because of the validation procedures which we will introduce in section ‘Stratum validation strategies’. Because of the validation and iterative re-matching it may occur that a group contains more than one case and more than one control.

We also note that one might consider multi-objective matchings similar to [47,48], in order to obtain groups already in the initial steps of the matching. However, the removal and repeated matching of invalid pairs due to the stratum validation completely compensates for the optimality-advantage of more sophisticated methods. We therefore stick to our computationally more efficient method.

#### Agglomerative hierarchical clustering

The Hungarian Method can be utilized as a basic building block for agglomerative hierarchical clustering. This is called the *Hungarian Clustering Algorithm* [37]. The algorithm is capable to cluster non-convex data sets. The widely used K–Means- or EM-based clustering approaches, for instance, have difficulties with such data sets. It is also robust to noisy data due to the hierarchical nature that prevents fast propagation of clustering errors. The number of clusters is intrinsically found as part of the process, while the performance of the algorithm is quite competitive to other clustering methods. A description of our implementation of the Hungarian Clustering Algorithm is given in Additional file 1: Appendix B. For comparison to other approaches like the spectral clustering we refer to [37].

### Stratum validation strategies

The Hungarian Method provides an optimal matching, i.e. a matching with an extremal sum of weights over all matches. It does not guarantee that in each matched pair both individuals are actually very close. In particular for samples with strongly asymmetric strata in the study sample, inter-stratum matches occur due to stratum-wise excesses of cases or controls. Therefore, we have to perform a “quality check” on the matching, and pairs that fail the validation are removed from the study sample. For the groupwise matchings, removed individuals may iteratively be re-matched and validated again until they are matched or identified as not matchable. In the following we propose two strategies to perform such a stratum validation.

#### Intra-cluster matching

Preceding to the case-control matching, cluster affiliation is obtained by a clustering algorithm (e.g. section ‘Agglomerative hierarchical clustering’). Then the matching is performed in each cluster separately. This enforces case-control pairs/groups to originate from the same stratum. The idea to perform a within-cluster matching after obtaining cluster information is also given in [19].

#### Vicinity check

For the vicinity check we adopt and extend the idea of the parameter *T*_*all*_ from the Hungarian Clustering Algorithm, presented in Additional file 1: Appendix B. In contrast to the purpose of clustering, for the problem at hand we have a bias in the distribution of individuals due to the case-control binary trait. For a case, we only consider neighboring controls and vice versa. Therefore, we relax the vicinity parameter of eq. (B.4) (Additional file 1) to

(2) $$ T_{cc}= \ln (|O| |A|)~.  $$

The Hungarian Method returns a matching *M*⊂*E* with case-control pairs (*x*,*y*). For a found pair we count for both individuals, using *s*_*ij*_, how many pairings with a higher IBS-value can be found in the data set. Let $t_{x}^{(y)}$ be the number of controls that have a higher weight to the case *x* than the control *y* and let $t_{y}^{(x)}$ be the number of cases that have a higher weight to the control *y* than the case *x*, that is $ t_{x}^{(y)} = \text {count} \{ w(x,j) > w(x,y) : \, j = 1\ldots |O| \}$ and $ t_{y}^{(x)} = \text {count} \{ w(i,y) > w(x,y) : \, i = 1\ldots |A| \}$. Then if the vicinity condition

(3) $$ \left(t_{x}^{(y)}<T_{cc} \wedge t_{y}^{(x)}<T_{cc}\right) ~\vee~ t_{x}^{(y)}=0 ~\vee~ t_{y}^{(x)}=0  $$

is fulfilled the pair is valid, otherwise the pair is removed. The parameter *T*_*cc*_ does guarantee close relatedness in the sense that it removes pairs which are too far apart compared to the vicinities of the individuals. On the other hand it does not enforce a pair belonging to the same stratum. We expect this approach to be robust in the presence of strong asymmetric strata, in particular if strata are overlapping. In other words, the passing condition eq. (3) checks for a well-defined abundance of possible better mates (first term) or if there are no better mates at all (remaining terms).

### Matching Cochran-Armitage Trend test

#### Squared test: MCAT^(2)^

In genetic association studies, the usually employed formula for the *Cochran-Armitage trend* (CAT) test statistic [38,39] with the co-dominant model, *t*=(0,1,2), for a particular SNP is given by

(4) $$ \begin{aligned} X_{T^{2}} \,=\, \frac{T^{2}}{\sqrt{\text{Var}(T^{2})}} \,=\, \frac{N}{n_{co}n_{ca}} \frac{\left[n_{co}(n_{22}+2n_{23}) - n_{ca}(n_{12}+2n_{13})\right]^{2} }{N(n_{\star2}+4n_{\star3}) - (n_{\star2}+2n_{\star3})^{2}} ~. \end{aligned}  $$

The nomenclature for this equation is defined in Table 1. Note that only individuals with non-missing genotypes for the particular SNP can be considered. For large population samples the test statistics asymptotically converges to a squared standard normal distribution or chi-squared distribution with one degree of freedom, ${\lim }_{{N\rightarrow \infty }} X_{T^{2}} \sim \mathcal N(0,1)^{2} = {\chi ^{2}_{1}}$. Let us now generalize the CAT test to provided structures in terms of pairs, group or clusters, which we will summarize *units* in the following. For that, we calculate the test statistic in each unit separately and add up all test statistics to a joint test statistic. Due to the square in the numerator of eq. (4), an interchange of the allele frequencies between cases and controls would contribute equally. Therefore, we have to weight each statistic with a sign corresponding to the relation of allele frequencies of cases and controls

(5) $$ Y_{T^{2}} = \left|\sum_{i=1}^{M} \text{sgn} \left(f_{ca}^{(i)} - f_{co}^{(i)} \right) X_{T^{2}}^{(i)} \right| ~,  $$

**Table 1:** **Genotypic contingency table for risk/reference allele** ***a*** **/** ***A***

	***AA***	***aA***	***aa***	**Sum**
Controls	*n*_11_	*n*_12_	*n*_13_	*n*_*co*_
Cases	*n*_21_	*n*_22_	*n*_23_	*n*_*ca*_
Sum	*n*_⋆1_	*n*_⋆2_	*n*_⋆3_	*N*

where *M*=|units| is the number of units. The risk allele frequencies of the unit *i* are given by $f_{\textit {co}}^{(i)} = (2n_{11} + n_{12})/n_{\textit {co}}$ for controls and $f_{\textit {ca}}^{(i)} = (2n_{21} + n_{22})/n_{\textit {ca}}$ for cases. Due to the signed nature of the contribution per unit in eq. (5), the test statistic $Y_{T^{2}}\phantom {\dot {i}\!}$ cannot be expressed by a ${\chi ^{2}_{n}}$-distribution, and we will need to employ resampling simulations in order to calculate P-values. In the following, we will call the test statistics $Y_{T^{2}}\phantom {\dot {i}\!}$ the *squared Matching Cochran-Armitage Trend* (MCAT^(2)^) test.

#### Linear test: MCAT^(1)^

Let us now consider the linear version of the CAT test statistic with the co-dominant, *t*=(0,1,2), model

(6) $$ \begin{aligned} {}X_{U} \!= \frac{U}{\sqrt{\text{Var}(U)}} = \sqrt{\frac{N-1}{n_{co}n_{ca}}} \frac{n_{co}(n_{22}+2n_{23}) - n_{ca}(n_{12}+2n_{13}) }{\sqrt{N(n_{*2}+4n_{*3}) - (n_{*2}+2n_{*3})^{2}}} ~. \end{aligned}  $$

For large population samples the distribution function of the test statistic converges to a standard normal distribution, ${\lim }_{N \rightarrow \infty } X_{U} \sim \mathcal N(0,1)$. Note, that the finite population correction factor $\sqrt {(N-1)/N}$ compared to eq. (4) is necessary in order to guarantee unbiasedness of the expectation value $E({X_{U}^{2}})$ and to fix Var(*X*_*U*_)=1 for all sample sizes *N*. It is straightforward to verify this factor by calculating Var(*U*) using the multivariate hypergeometric distribution. Let us now generalize the CAT test to a provided sample structures. We again calculate the test statistic in each unit separately and add up all test statistics to one joint test statistic

(7) $$ Y_{U} = \sum_{i=1}^{M} X_{U}^{(i)} ~.  $$

The resulting test statistic for large *M* is asymptotically normal distributed with variance *M*, ${\lim }_{M \rightarrow \infty } Y_{U} \sim \mathcal N(0,M)$. Note, that the convergence is quite fast, therefore it is reasonable to employ this test for more than a dozen pairs/groups while the variance is finite. To be more precise, the test can be transformed to a standard normal distribution where the test statistic scales with $1/\sqrt {M}$. Likewise, considering large clusters, the test statistic is normally distributed for small *M* with large numbers of individuals per unit *N* ^(*i*)^, ${\lim }_{N^{(i)} \rightarrow \infty, \forall i \in M} Y_{U} \sim \mathcal N(0,M)$. In the following, we will call the test statistics *Y*_*U*_ the *linear Matching Cochran-Armitage Trend* (MCAT^(1)^) test compared to the squared test MCAT^(2)^.

### Determination of P-values

#### (M)CAT tests with resampling simulation

We obtain P-values for the CAT and MCAT^(2)^ tests by utilizing resampling simulations on the basis of within-unit – i.e. within-pair, -group or -cluster – permutation of the case-control trait. The P-values are determined by the fraction of simulations, where the resulting test statistic is equal to or more extreme than the test statistic of the original set. We also allow adjustment for multiple testing by employing the minP approach [49], that has previously been used in the context of pathway association analysis [50]. One considerable strength of the minP approach is that it allows to avoid nested simulations.

#### Regression models with structure covariates

Regression models with structure covariates provide useful tools to perform stratified analyses without employing resampling simulations. For case-control studies we may employ logistic regression (LR) and for quantitative trait studies linear regression. Population structure covariates are obtained by calculating principal components using MDS or PCA. The P-values are calculated using the likelihood ratio test.

### Simulation of stratified population samples

The simulation study is based on the genotype data of the 14 population samples from 4 continents of the 1,000 Genomes Project phase 1 integrated release [51] (data access Mar 2012), where we use SNPs from just chromosome 22. Since some of the samples are rather small, we first create larger samples in the following way: for each population, we estimate allele frequencies from a set of 500,000 SNPs and local 2-SNP-haplotype frequencies from the original data. These frequencies serve as parameters for the simulation of data sets with 4,000 individuals for each population. Thus, our simulated data meet the original data with respect to allele frequency distribution and pairwise LD, but do not capture higher order LD. We feel that this potential loss of information is compensated by the fact that it is considerable to extend sample sizes beyond the original sample sizes of the 1,000 Genomes project. The TSI population, for instance, is represented by a sample of only 14 individuals so it would not have been possible to include it in the simulation without our treatment. Inflating populations from a small source does over-estimate the abundance of monomorphous SNPs. Therefore, we will remove SNPs that are monomorphous in any population in our following simulations.

## Results and discussion

### Simulated multi-population study

#### *H*_0_ simulation

From each simulated population sample of section ‘Simulation of stratified population samples’, we randomly select cases and controls under the null hypothesis of no association within each population stratum. After this, we merge the genotype data of the strata, thereby producing population stratification, and discard the population information for further analysis. In this way, we simulate about 44,900 SNPs and 1,845 individuals, where the distribution of cases and controls from each stratum is strongly asymmetric and is listed in Table 2. Thus we mimic over/under-sampling of cases from different strata, thereby generating stratified data sets. We create each simulated data set tenfold in order to ensure that we do not obtain an accidentally outlying set, and will state the mean and standard error of the inflation factor *λ* and the false-positive rate *f*_*p*_.

**Table 2:** **Distribution of the 1,845 individuals (967 controls, 878 cases) from 14 distinct ancestries**

**Ancestry**	**AFR**			**AMR**			**ASN**			**EUR**				
	**ASW**	**LWK**	**YRI**	**CLM**	**MXL**	**PUR**	**CHB**	**CHS**	**JPT**	**CEU**	**FIN**	**GBR**	**IBS**	**TSI**
ncontrols	85	96	66	30	88	36	130	66	45	31	92	120	9	73
ncases	42	48	66	60	44	73	65	133	88	61	46	60	18	74

In Table 3 we show the results of a single-marker analysis using the CAT, the MCAT^(2)^ and the MCAT^(1)^ tests with all combinations of structuring (pairs, groups, cluster) and validation (pre-cluster, vicinity) methods. We compare these results with the logistic regression test employing 7, 14 and 28 structure covariates in terms of principal components obtained by multidimensional scaling. For the logistic regression tests the P-values are obtained by the likelihood ratio test, for the MCAT^(1)^ test by the asymptotic normal distribution and for the CAT and MCAT^(2)^ tests by resampling simulations (section ‘(M)CAT tests with resampling simulation’) with 99,999 cycles. False-positive rates are given by the fractions of SNPs that show a P-value that is smaller than a nominal error level of *α*=0.05. The inflation factors are calculated from the median of the test statistics divided by the expected median value of a ${\chi ^{2}_{1}}$-distributed statistic (≈0.456). For the MCAT^(1)^ test, in order to have one standardized definition of the inflation factor, we use the squared test statistic of eq. (7), ${Y_{U}^{2}}/M$. The initial inflation for the asymptotic CAT test yields *λ*=1.990 and *f*_*p*_=0.167, while a resampling simulation of all individuals shows no significant change on this *λ*=1.900 and *f*_*p*_=0.163. This indicates a sensible amount of stratification in the population sample.

**Table 3:** **H0-simulation: inflation factor and false-positive rates**

**Test**		**Units**	**Validation**	**N**	***λ***	***σ***_***λ***_	***f***_***p***_	${\sigma _{f_{p}}}$
CAT	AT	–	–	1845	1.990	0.013	0.167	0.001
CAT	RSU	All	–	1845	1.900	0.013	0.163	0.002
CAT	RSU	Pairs	Cluster	1322 (661p)	0.853	0.008	0.044	0.001
CAT	RSU	Pairs	Vicinity	1254 (627p)	0.846	0.007	0.044	0.001
CAT	RSU	Groups	Cluster	1845 (661g)	0.921	0.006	0.047	0.001
CAT	RSU	Groups	Vicinity	1845 (627g)	0.921	0.009	0.046	0.001
CAT	RSU	Clusters	–	1845 (14c)	0.918	0.006	0.046	0.001
MCAT^(2)^	RSU	Pairs	Cluster	1322 (661p)	0.832	0.008	0.044	0.001
MCAT^(2)^	RSU	Pairs	Vicinity	1254 (627p)	0.828	0.010	0.043	0.001
MCAT^(2)^	RSU	Groups	Cluster	1845 (661g)	1.005	0.011	0.050	0.001
MCAT^(2)^	RSU	Groups	Vicinity	1845 (627g)	1.001	0.011	0.050	0.001
MCAT^(2)^	RSU	Clusters	–	1845 (14c)	1.004	0.009	0.050	0.001
MCAT^(1)^	AT	Pairs	Cluster	1322 (661p)	1.007	0.009	0.050	0.001
MCAT^(1)^	AT	Pairs	Vicinity	1254 (627p)	1.007	0.011	0.050	0.001
MCAT^(1)^	AT	Groups	Cluster	1845 (627g)	1.007	0.012	0.051	0.001
MCAT^(1)^	AT	Groups	Vicinity	1845 (627g)	1.008	0.010	0.050	0.001
MCAT^(1)^	AT	Clusters	–	1845 (14c)	1.000	0.007	0.050	0.001
LRmds	LRT	7 PCs	–	1845	1.201	0.019	0.078	0.001
LRmds	LRT	14 PCs	–	1845	1.006	0.009	0.051	0.001
LRmds	LRT	28 PCs	–	1845	1.017	0.009	0.052	0.001

Given are means and standard errors of the inflation factor *λ* and false-positive rates *f*_*p*_ from ten iterations of 1845 individuals and ∼44,900 SNPs. The nominal error level is *α*=0.05. The abbreviations in the second column are: AT asymptotic test, RSU resampling simulation within units (99,999 cycles), LRT likelihood ratio test. Column N shows the number of individuals included and in brackets the number of pairs *p*, groups *g* and clusters *c*.

While the naive CAT test with resampling within units over-compensates for stratification effects, both MCAT tests in general perform much better. The groupwise matchings show overall good results in terms of inflation (*λ*≈1) and consumption of the statistical level (*f*_*p*_≈0.05). We find that both validation methods (pre-cluster and vicinity) work well with the groupwise matching. In Table 3 the number of found groups for the vicinity validation is smaller than for the clustering validation. Thus, the vicinity validation is more stringent than the clustering validation. The clustering approach was able to detect all 14 populations in nearly all of the ten samples, sometimes deviating by one. The pairwise matchings reduce the sample to become equal in the number of cases and controls and remove the least fitting individuals. For that reason it is comprehensible that it tends to produce samples that are deflated (*λ*<1, Table 3).

The MDS approach also yields very good results if we provide the correct number of populations (LRmds14, *λ*=1.006). However, underestimating the number does only partially correct for population stratification (LRmds07) and also overestimating the number (LRmds28) shows a slow increase of the false-positive rate. We think the latter may happen due to cancellation of redundant components, which leads to a drop-off in the ability to correct for stratification.

Computation time of the resampling simulation (99,999 cycle, 44,900 SNPs) on the MCAT^(2)^ test with groupwise matching takes about 450 minutes on a single used core of an Intel®; Xeon®; E5540 CPU with 2.53GHz. Our implementation supports parallelization, which reduces the real time correspondingly.

#### Power simulation

We create a series of data sets by moving along the simulated chromosome. A particular encountered SNP is assigned to be associated and we assume a relative disease risk of 1.5 under a multiplicative model. Based on this model assumption and on strata-specific baseline allele frequency we simulate cases and controls from each of the 14 population samples of section ‘Simulation of stratified population samples’ with the distribution given in Table 2. In this way, we mimic over/under-sampling of cases from different strata, thereby generating stratified data sets that each contain one true SNP association. By construction, the biased sampling from different strata may blur the true association effect in the joint sample. In total, our procedure yields 11,010 data sets with the case-control status under this model. We use 10,000 SNPs for the matching and keep a gap of at least 1,000 SNPs to the analyzed SNP to guarantee that there is no LD between SNPs that are used for the matching and the associated SNP. We determine the P-values as described in section ‘*H*_0_ simulation’. On the results of the power study we perform genomic control: from the P-values we calculate the test statistics by an inverse *χ* ^2^-distribution, then correct those test statistics by the inflation factors of Table 3, and finally calculate again the P-values according to a *χ* ^2^-distribution.

**Figure 1:**
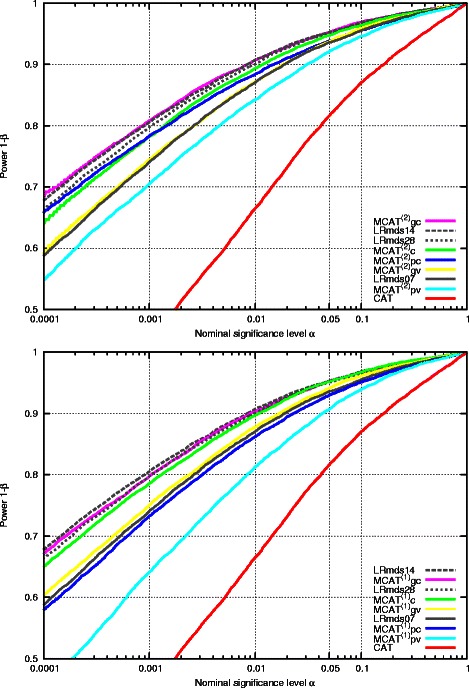
**ROC curve.** Power simulation of 11,010 SNPs, 1845 individuals. P-values are corrected via genomic control using the corresponding inflation factor from the simulation under *H*_0_. The abscissas are given in logarithmic scale. The upper plot compares the MCAT^(2)^ with the principal components approach. The lower plot shows the asymptotic test MCAT^(1)^. The subscripts in the legend denote the employed structures: clusters *c*, groups (pairs) with cluster validation *gc* (*pc*) and groups (pairs) with vicinity validation *gv* (*pv*).

In Figure 1 we illustrate the power vs. the nominal error level for both MCAT^(2)^ and MCAT^(1)^ test and the logistic regression test with covariates. In Table 4 we list the power for three selected nominal error levels (0.01, 0.001, 0.0001) for all employed tests. We observe that the unstructured association testing (CAT - red line) is fully outperformed by all structured association testing methods. The logistic regression model with an over-estimated count of MDS covariates (LRmds28 - dashed gray line) performs considerably weaker than the model with the optimal count (LRmds14 - solid gray line). The naive CAT tests, with resampling simulations performed within units, drop off rapidly for already very large nominal levels (Table 4). Both MCAT tests (colored lines) for the groupwise matching and the cluster are competitive with LRmds14. Reducing the sample size to matched pairs (blue and turquoise lines) reduces power considerably. We conclude, that groupwise matching should definitely be favored over pairwise matchings.

**Table 4:** **Power simulation: power vs. selected nominal levels for all strategies**

			**Power 1−** ***β***			
**Test**		**Units**	**Validation**	***α*** **=0** ***.*** **01**	***α*** **=0** ***.*** **001**	***α*** **=0** ***.*** **0001**
CAT	AT	–	–	0.665	0.450	0.271
CAT	RSU	Pairs	Cluster	0.881	0.782	0.662
CAT	RSU	Pairs	Vicinity	0.846	0.710	0.558
CAT	RSU	Groups	Cluster	0.809	0.705	0.594
CAT	RSU	Groups	Vicinity	0.833	0.729	0.607
CAT	RSU	Clusters	–	0.808	0.704	0.597
MCAT^(2)^	RSU	Pairs	Cluster	0.884	0.783	0.658
MCAT^(2)^	RSU	Pairs	Vicinity	0.823	0.705	0.549
MCAT^(2)^	RSU	Groups	Cluster	0.907	0.808	0.685
MCAT^(2)^	RSU	Groups	Vicinity	0.872	0.745	0.591
MCAT^(2)^	RSU	Clusters	–	0.894	0.782	0.641
MCAT^(1)^	AT	Pairs	Cluster	0.862	0.733	0.579
MCAT^(1)^	AT	Pairs	Vicinity	0.813	0.641	0.454
MCAT^(1)^	AT	Groups	Cluster	0.903	0.797	0.671
MCAT^(1)^	AT	Groups	Vicinity	0.878	0.751	0.603
MCAT^(1)^	AT	Clusters	–	0.898	0.786	0.650
LRmds	LRT	7 PCs	–	0.871	0.741	0.588
LRmds	LRT	14 PCs	–	0.908	0.805	0.678
LRmds	LRT	28 PCs	–	0.901	0.798	0.664

11,010 SNPs, 1845 individuals, nominal error level *α* and power 1−*β*. 10,000 independent SNPs were used to obtain structure information. The abbreviations in the second column are: AT asymptotic test, RSU resampling simulation within units (99,995 cycles), LRT likelihood ratio test. Column N shows the number of individuals included and in brackets the number of pairs *p*, groups *g* and clusters *c*.

### Stratification from fine scale population structure in an Alzheimer’s disease sample

We apply the methods to a GWAS on Alzheimer’s disease (AD) which uses the Illumina®; Omni1M micro-array. The study has been described in [52]. AD patients have been recruited within the German Dementia Competence Network and at the interdisciplinary memory clinic of the Department of Psychiatry and Department of Neurology at the University Hospital in Bonn, Germany. Diagnosis was established according to NINCDA-ADRDA criteria [53]. After application of standard quality control, genotypes of 850,612 SNPs are available for 631 cases and 1,080 controls. We exclude a ±1 MB region surrounding the APOE gene, since its well-established strong association with AD would interfere with the inflation factor, and SNPs with a minor allele frequency (MAF) below 0.02. Analysis with the CAT test yields a genome-wide inflation factor of *λ* ^AD^=1.087 for the unstructured analysis, indicating residual inflation.

In order to clarify if this inflation is likely due to population stratification we proceed as follows: we aim to identify SNP loci with an allele frequency distribution that varies according to the geographic origin within Europe utilizing information from the 1,000 Genomes Project data [51]. In detail, we filter the 1,000 Genomes data set for MAF >0.01 and compare SNPs from the CEU sample with the FIN, GBR and TSI sample, respectively, in a case-control fashion. The respective genome-wide inflation factors are $\lambda ^{\text {1kG}}_{\text {CEUvsFIN}}=2.110$, $\lambda ^{\text {1kG}}_{\text {CEUvsGBR}}=1.127$ and $\lambda ^{\text {1kG}}_{\text {CEUvsTSI}}=1.563$, which demonstrates the presence of a considerable portion of SNPs that differ in allele frequency between the subsamples in [51]. For each of the three comparisons, we retain the SNPs with *p*<0.05 as a pre-selection for our AD-GWAS study. These CEUvsFIN, CEUvsGBR and CEUvTSI SNP-sets contain false-positive SNPs but are also enriched for SNPs with group-specific genotype distribution. Indeed, when we restrict the analysis of our independent AD-GWAS to these SNP-sets, we obtain inflation factors that were markedly higher ($\lambda ^{\text {AD}}_{\text {CEUvsFIN}}=1.097$, $\lambda ^{\text {AD}}_{\text {CEUvsGBR}}=1.101$, $\lambda ^{\text {AD}}_{\text {CEUvsTSI}}=1.132$) compared to the inflation factor of the entire GWAS (*λ* ^AD^=1.087). Our interpretation is that the group-specific SNPs follow a genotype distribution that is correlated with the geographic location within Germany and particularly migration and therefore cause moderate population stratification within our study.

Next, we investigate how the observed population stratification is accounted for by our and by well-established stratification methods. We have determined that for the 13 leading covariates the inflation factor from the LR-MDS [54] test has a minimum (*λ*=1.032). With a more increasing count of used components we observe a slight but steady increase in the inflation factor. A test for significance for each covariate, employed on each single SNP and corrected for multiple testing on 50 employed principal components, revealed an overall impact by the components 3, 4, 5, 9, 10 & 13. We repeated the analysis with PCA components in combination with LR using the PLINK 1.9 software [55] resulting in an optimum of 7 leading components (*λ*=1.021) and the significant components 2, 4, 5, 6, 18 & 19. For comparison we also employed the *mixed linear model based association* (MLMA) test of the GCTA software [56].

In Table 5 we show the ranking association results, that we obtain from the analysis each without adjustment for population stratification (*λ*=1.087), our three comparison methods, and with the groupwise matching with the vicinity validation for the MCAT^(2)^ test (*λ*=1.044). The top 5 hits of all methods are marked bold. In most cases, the strength of association of the results from the unstratified analysis drops with all methods for stratification. There is, however, a noteworthy exception among the top results of the linear regression model with principal component covariates. rs3094078 (*f*=0.120), located within the major histocompatibility complex (*MHC*) on chromosome 6, reaches *p*=6·10^−8^ with the MDS- and *p*=5·10^−7^ with the PCA-approach. In contrast, the unstratified analysis only shows a P-value of *p*=0.003. The groupwise stratification analysis likewise shows a much less impressive level of significance with *p*=0.013 which is supported by the MLMA test (*p*=0.031).

**Table 5:** **Comparison of top ranking associations between different stratification methods**

**ID**	**chr:pos**	**P**_**CAT**_	$\text {P}_{\text {LR-mds13}}^{\text {[54]}}$	$\text {P}_{\text {{LR-pca07}}}^{\text {[55]}}$	$\text {P}_{\text {{MLMA}}}^{\text {[56]}}$	**P**_**MCAT-gv**_
		***λ*** **=1** ***.*** **087**	***λ*** **=1** ***.*** **027**	***λ*** **=1** ***.*** **021**	***λ*** **=0** ***.*** **998**	***λ*** **=1** ***.*** **044**
rs13320534	3:46171700	**7.35e-8**	**2.32e-7**	**5.22e-7**	**2.17e-7**	**4.63e-6**
rs936939	3:45986623	**9.85e-7**	**1.36e-6**	**2.20e-6**	**2.40e-6**	1.55e-5
rs9967637	19:57250898	**1.04e-6**	6.50e-6	1.24e-5	**1.96e-6**	4.96e-5
rs17650960	15:27999442	**1.84e-6**	7.93e-5	9.67e-5	5.33e-5	3.81e-5
rs10902222	11:810882	**1.89e-6**	2.76e-5	1.14e-5	1.04e-5	4.16e-5
rs1992102	3:21280562	2.80e-6	**1.55e-6**	**1.22e-6**	**2.93e-6**	2.38e-2
rs2962492	5:39568609	7.54e-6	**3.72e-6**	**4.19e-6**	**7.08e-6**	**1.08e-6**
rs4673251	2:204114244	1.70e-5	6.90e-6	1.13e-5	2.03e-5	8.57e-2
rs16844699	3:103879674	4.00e-5	6.04e-5	6.04e-5	3.50e-4	**1.50e-5**
kgp9470129	3:141298124	5.30e-5	3.70e-5	2.67e-5	2.20e-5	**1.34e-5**
rs8073498	17:7569698	1.32e-4	1.66e-3	1.08e-3	6.90e-4	**1.36e-5**
rs3094078	6:30224970	3.16e-3	**5.85e-8**	**5.00e-7**	3.05e-2	1.28e-2

The indices of the P-values refer to the type of test: CAT test without any stratification method, LR-mds13/LR-pca07 for logistic regression with 13 MDS/7 PCA covariates, MLMA stands for mixed linear model association and MCAT-gv for our modified CAT test with group unit and vicinity validation.

It turns out that the first PC is highly correlated with rs3094078, Pearson correlation *r*(PC1,SNP)=0.80, while the correlations between SNP and case-control status, *r*(SNP,phen)=−0.07, and case-control status with first PC, *r*(phen,PC1)=−0.02, are negligible. There is no association of PC 1 with the trait *P*=0.6. Employing only PC 2-7 in the regression model, we obtain CI _95*%*_(*β*)=[0.17;0.62] and *P*=6.1·10^−4^ for rs3094078. Adding PC 1 changes this to CI _95*%*_(*β*)=[0.59;1.36] and *P*=5.0·10^−7^, while PC 1 also becomes significant. Using MDS covariates leads to virtually identical results. We think it is possible, that an accidental correlation of the leading component with the SNP has boosted a false-positive signal. In this context, external knowledge does not convincingly support association of rs3094078 with AD. The International Genomics of Alzheimer’s Project (IGAP) recently published results of a joint meta-analysis [57]. The published list of IGAP SNPs with *p*≤0.001, contains a region 147 kb to 207 kb upstream from our result with 75 SNP signals with P-values in the range [0.69,9.93]·10^−4^, but no signals that come close to genome-wide significance are listed within ±2 MB of our signal. IGAP actually found rs9271192 from HLA-DRB1 from the *MHC* to be associated with AD at genome-wide significance. This signal, however, resides more than 2.3 Mb away from rs3094078. According to the CEU [51] reference data, rs3094078 and rs9271192 are not in LD (*r* ^2^=0.001). In summary, the IGAP analysis does not strongly support association of our top signal from analysis using principal components with AD and suggests that the results of the matching stratification methods are more realistic.

## Conclusions

We presented a framework which allows for structured association testing of arbitrarily complex population samples. It is based on pairwise/groupwise matchings (section ‘Structuring strategies’) obtained utilizing the assignment algorithm in combination with validation methods (section ‘Stratum validation strategies’). In addition, we applied an agglomerative hierarchical method that allows clustering (section ‘Agglomerative hierarchical clustering’) without the need of any model assumptions to be made on the underlying sample. P-values for the CAT and the MCAT ^(2)^ tests (section ‘Squared test: MCAT ^(2)^’) were obtained by simulations via permutation testing, while the MCAT ^(1)^ (section ‘Linear test: MCAT ^(1)^’) test is asymptotically normal distributed. We found that the CAT test generally is insufficient for the analysis with population structure. The new MCAT tests shows considerable improvements over the CAT test for analysis of such samples and is competitive with principal components approaches in logistic regression models.

The pairwise matching reduces the sample (forfeits statistical information) and tends to pick an over-deflated subsample of asymmetric samples (inflation factor <1). We strongly advise to utilize it for analysis of discordant sib-pair only. If it is possible to detect a clear cluster structure without substructure clustering performs optimal. Also, clustering does not rely on case-control information, therefore it can be used with either binary or quantitative traits. If clusters and substructure are present, the groupwise matching with cluster validation is a good choice. In the case that no cluster-structure can be revealed, we advise to apply the groupwise matching with vicinity validation.

We found that, for principal components approaches, the number of needed PCs has to be estimated relatively accurately in order to obtain not-inflated analysis results. It has been claimed [58] that the first ten principal components explain the majority of variance attributed to population structure. In contrast there is a study [13], where ten principal components are insufficient to expose population substructure, which consequently leads to a spurious association of the lactate gene *LCT* with body height. For the same study GC was not able to correct for stratification [13] and also clustering approaches failed to detect population substructure [59]. It is useful to verify results by employing different approaches such as ours for a cross check. In section ‘Stratification from fine scale population structure in an Alzheimer’s disease sample’ we presented another example of a possibly false-positive associated SNP of the MHC region with Alzheimer’s disease using the principal component approaches. We illustrated that the finding is not supported by our new developed methods and has neither been found in [57] nor been approved by the MLMA test.

We wish to point out that matching methods are of particular interest for models, for which the inclusion of covariates is not possible or hard to model. We empirically found that about 7,500 common variants are sufficient to calculate the genetic similarity scores, from which the matchings are derived. Thus the score can be evaluated both genome-wide and window-wise. The latter is of particular interest for finding region-specific matches for rare-variants analysis, which was proposed in [3]. The application and extension of the provided methods to rare-variants analysis [5] will be a topic for future investigations.

All methods described are implemented in INTERSNP [60], which is a stand-alone C/C++ software, freely available under the GNU license, that was originally developed for genome-wide interaction analysis [61]. The software is fully compatible with all PLINK [54] input file formats. All matching procedures can be conducted genome-wide and are documented [60].

## Additional file

Additional file 1
**Appendices A and B.**
