# Supplementary material for: Novel genetic matching methods for handling population stratification in genome-wide association studies
Source: BMC Bioinformatics. 2015 Mar 14;16:84. doi: 10.1186/s12859-015-0521-4 (PMC4367953; doi:10.1186/s12859-015-0521-4)
Supplement: Additional file 1 — Appendices A and B. [file 12859_2015_521_MOESM1_ESM.pdf]

ONLINE-ONLY SUPPLEMENTARY DATA

Novel genetic matching methods for handling population stratification in genome-wide association studies

André Lacour[1*], Vitalia Schüller[1], Dmitriy Drichel[1], Christine Herold[1], Frank Jessen[1,3], Markus Leber[2], Wolfgang Maier[3], Markus M. Noethen[4], Alfredo Ramirez[3], Tatsiana Vaitsiakhovich[2] and Tim Becker[1,2]

Appendix A: Binary coding

Here we discuss our approach of storing genotype data and obtaining elements of IBS matrices and contingency tables. For each individual we employ one array of an integer data type with the largest word length l available for the computation architectures (for both `C/C++` and hardware currently $l = 64\,\text{bit}$). The size n of that array has to be sufficiently large to provide one bit for each of N SNPs, thus $n = \lceil N/l \rceil$. This structure is needed once for each genotype and the missingness of genotyping information $\{\text{gt0}, \text{gt1}, \text{gt2}, \text{missing}\}$, thus four times in total. In short, we provide a multidimensional array of dimension `array(M)(n)(4)`, where M is the number of individuals. In this way, for every individual and every SNP four bits are provided. The bit of the genotype, corresponding to the actual genotype at a given SNP and individual, is set to 1, and 0 for the other three. This kind of storage saves 50% of memory compared to one-byte-per-genotype storage. It is possible to save another 25% of memory, at the cost of a small additional computational burden, by omitting the missing genotyping information and replace its access, where needed, by $\sim(\,\text{gt0}\,|\,\text{gt1}\,|\,\text{gt2}\,)$. Calculation of the IBS value between two individuals can now be performed by comparing, according to

$$s_{ij} = 1 - \frac{1}{2N} \sum_{k=1}^{N} |g_{ik} - g_{jk}| \;, \tag{A.1}$$

where N is the number of loci successfully genotyped in both individuals and g_{ik} is the nominal genotype $\{0, 1, 2\}$ (number of expressed minor alleles) for individual i at locus k, the genotypes of 64 SNPs at once using binary operations. Then the information of those SNPs is added by calculating the horizontal checksum by employing modern versions of the Hamming weight method [1].

*Correspondence: andre.lacour@dzne.de
[1]German Center for Neurodegenerative Diseases (DZNE), Sigmund-Freud-Str. 25, 53127 Bonn, Germany
Full list of author information is available at the end of the article

In the same manner, by transposing the first two dimensions of the array, yielding `array(N)(m)(4)` with $m = \lceil M/l \rceil$, we are able to calculate the elements of contingency tables, minor allele frequencies and missing rates with a tremendous gain in computational performance compared to naïve integer evaluations.

Appendix B: The Hungarian Clustering Algorithm

Here we describe the algorithm adapted from [2]. The basic idea of the algorithm is the correspondence between the assignment problem and the cycle cover problem. The cycle cover problem seeks an extremized-weight subset of edges that constitutes a union of cycles, while each vertex belongs to exactly one cycle. To apply this idea we consider a complete *weighted bipartite graph* $G(V, E, w)$, with *vertices* $V = O \cup A$, in which $O \cap A = \emptyset$, *edges* $E \subseteq V \times V$ and *weights* $w : E \to \mathbb{R}$. The edges are weighted with the pairwise IBS values of the individuals $w(i, j) = s_{ij}$, for $i \neq j$, and the diagonal weights are set to zero, $w(i, i) = 0$, to represent the non-existence of loops. Note, that the set of edges for this problem is $E \subseteq V \times V$, where for the assignment problem it was $E \subseteq O \times A$.

Note, that the authors of that work considered minimized distances, whereas, in our work, we consider the maximized weights. In a given iteration step of the algorithm we have k clusters, with l_i elements each, $C_i = \{c_{i,1}, \ldots, c_{i,l_i}\}$ where $i = 1 \ldots k$ and $V = \bigcup_{i=1}^{k} C_i$.

0 regard every point as a single cluster, $k = n$ and $C_i = \{i\}\ \forall i = 1, \ldots, k$.

1 Compute the weight matrix $w(C_i, C_j)$ for all $1 \leq i, j \leq k$ according to the description given in the paragraph below.

2 Apply the Hungarian Method to find the optimal cycle cover in the complete weighted bipartite graph with vertices $\{C_1, \ldots, C_k\}$.

3 Update the number of clusters, k, to be equal to the number of cycles in the optimal cycle cover. New clusters are built up by the union of the old clusters that are connected by the cycles in the optimal cycle cover.

4 If the number of new clusters is less than the number of old clusters, go to step 1. Otherwise we are finished.

For **calculating the weight matrix** we employ the following procedure: let T be some fixed integral parameter.

1 Consider all clusters pairwise: take the maximal weight between points of both sets

$$w(C_i, C_j) = \max\{w(c_{i,r}, c_{j,s}) \,|\, r = 1 \ldots l_i,\ s = 1 \ldots l_j\}\ . \tag{B.1}$$

This is the maximized weight equivalent to the topological distance, which we will call *topological weight* in the following.

2 Let $c_{i,\hat{r}} \in C_i$ and $c_{j,\hat{s}} \in C_j$ be the points of maximal weight, $w(C_i, C_j) = w(c_{i,\hat{r}}, c_{j,\hat{s}})$. Let $t_i^{(j)}$ be the number of points inside cluster C_i that have a higher weight to the point $c_{i,\hat{r}}$ than to the cluster C_j:

$$t_i^{(j)} = \text{count}\{w(c_{i,\hat{r}}, c_{i,r}) > w(C_i, C_j) \,|\, r = 1, \ldots, l_i\}\ . \tag{B.2}$$

Then if

$$t_i^{(j)} > T \ \vee\ t_j^{(i)} > T\ , \tag{B.3}$$

set $w(C_i, C_j) = 0$. This regards two clusters as being too far apart to be combined.

3 Set $w(C_i, C_i) = 0$ which prevents a cluster to be matched as a singleton. If C_i has zero weight to all other clusters, $w(C_i, C_j) = 0\ \forall j = 1 \ldots k$, set $w(C_i, C_i) = \infty$. This marks a cluster as complete and the matching algorithm is forced to return a singleton cycle for this cluster.

The algorithm depends on the single parameter T, which is used to estimate whether clusters are too far apart. If the topological weight between two clusters is too large in comparison to the internal weights of at least one of the clusters we conclude that those clusters should not be merged. We agree with the authors of [2] that the algorithm is empirically not highly sensitive to the exact value of T. In case that the roughly expected abundance of clusters is much smaller than the size of the sample ($|C| \ll |V|$) we suggest to fix

$$T = \ln |V|\ . \tag{B.4}$$

The idea is quite plausible: the parameter has to be perceptibly smaller than the size of a cluster but still large enough to allow merging.

Author details

[1]German Center for Neurodegenerative Diseases (DZNE), Sigmund-Freud-Str. 25, 53127 Bonn, Germany. [2]Institut für Medizinische Biometrie, Informatik und Epidemiologie, Universität Bonn, Sigmund-Freud-Str. 25, 53127 Bonn, Germany. [3]Abteilung für Psychiatrie und Psychotherapie, Universitätsklinikum Bonn, Sigmund-Freud-Str. 25, 53127 Bonn, Germany. [4]Institut für Humangenetik and Life & Brain Center, Universität Bonn, Sigmund-Freud-Str. 25, 53127 Bonn, Germany.

References

1. Wegner P (1960). "A technique for counting ones in a binary computer", Communications of the ACM **3**, 322 (doi:10.1145/367236.367286).
2. Goldberger J, Tassa T (2008). "A hierarchical clustering algorithm based on the Hungarian method", Pattern Recognition Letters **29**, 1632–1638 (doi:10.1016/j.patrec.2008.04.003).
